# The Divergent and Conserved Expression Profile of Turtle *Nanog* Gene Comparing with Fish and Mammals

**DOI:** 10.3390/biology11091342

**Published:** 2022-09-12

**Authors:** Kaili Chen, Jianfei Xu, Wenzhuo Ban, Jiaming Tian, Zhiming Tan, Zhoukai Tang, Luo Lei, Wei Li, Xinping Zhu, Hongyan Xu

**Affiliations:** 1 Key Laboratory of Freshwater Fish Reproduction and Development, Ministry of Education, Key Laboratory of Aquatic Sciences of Chongqing, College of Fisheries, Southwest University, Chongqing 402460, China; 2 Key Laboratory of Tropical & Subtropical Fishery Resource Application & Cultivation of Ministry of Agriculture and Rural Affairs, Pearl River Fisheries Research Institute, Chinese Academy of Fishery Sciences, Guangzhou 510380, China

**Keywords:** *Nanog*, gene expression, reptile, oogenesis, spermatogenesis

## Abstract

**Simple Summary:**

Chinese soft-shell turtle (*Pelidiscus sinensis*) is a widely farmed aquatic reptile in China. It has of high nutritional, pharmaceutical, and economic value, but wild Chinese soft-shell turtle has been faced with an extreme decline of populations. Thus, it is of great significance to study the germ cells biology and develop the techniques for turtle breeding and genetic resource preserving. Nanog is a homeodomain-containing transcription factor, and it plays a vital role in maintaining the pluripotency of embryonic stem cells; there is a lack of reporting on the *Nanog* gene in reptile. In this study, the cDNA sequence and promoter of a *Nanog* gene was identified in *P. sinensis*, designed as *PsNanog*. *PsNanog* gene products were mainly expressed in gonads and were especially rich in ovary. Particularly, *PsNanog* mRNA and protein was exclusively expressed in germ cells in ovary and testis. Collectively, this is the first report on the gene structure and expression of *Nanog* in turtle, which may lay a foundation for further functional analysis of the *Nanog* in turtle, even in the reptiles. Further, PsNanog probably has the conserved and divergent function in regulating cell pluripotency and germ cell development in turtle, compared with other species, including teleosts and mammals.

**Abstract:**

Nanog is a homeodomain-containing transcription factor, and it plays a vital role in maintaining the pluripotency of embryonic stem cells. Nanog’s function has been well studied in many species. However, there is lack of reporting on the *Nanog* gene in reptile. Here, we identified a 1032 bp cDNA sequence of a *Nanog* gene in *Pelidiscus sinensis,* known as *PsNanog*. PsNanog has a highly conserved HD domain and shares a high identity with that of *Chelonia mydas* and the lowest identity with *Oryzias latipes*. Similarly, PsNanog presented a tight cluster with *C. mydas* Nanog, but was far from those of teleosts. Additionally, we cloned a length of 1870 bp *PsNanog* promoter. Dual luciferase assay showed that the DNA fragment of −1560 to +1 exhibited a high promoter activity. The RT-PCR and RT-qPCR results showed that *PsNanog* was predominantly expressed in ovary, and then in testis. The *in situ* hybridization and immunohistochemical analysis showed that PsNanog was expressed in the early primary oocytes and the cytoplasm of the cortical region of stage VIII oocytes in ovary, and distributed in most stages of germ cells in testis. Collectively, the results imply that *PsNanog* probably has the conserved function in regulating germ cell development across phyla and is also a pluripotent cell gene and expressed in germ cells, which is similar to that in teleosts and mammals.

## 1. Introduction

Nanog, a divergent transcription factor (TF), which nevertheless contains a highly conserved homeodomain (HD) in the process of species’ evolution, is identified in mammalian pluripotent cells and developing germ cells [1,2]. Nanog is considered to be one of the major TFs in pluripotent transcriptional network in mammals [3]. Thus, Nanog plays a vital role in autonomous self-renewal and maintaining the pluripotency of embryonic stem cells (ESCs) [1]. Theunissen et al. also showed that Nanog could reprogram the somatic cells back into the pluripotent state [4].

In mammals, Nanog has been studied in depth and it is considered to be a pluripotency marker strictly expressed in pluripotent cells, such as epiblast cells, primordial germ cells (PGCs), and ESCs of murine [1,2,5] and human origin [6]. In a vertebrate nonmammalian, chicken Nanog is expressed in the PGCs and blastoderm and then localized in epithelialized epiblasts of the embryo and the function in cESC is similar to mammals [7,8]. In urodele amphibian axolotl, Nanog is expressed in the axolotl animal cap and the study indicated that the mechanisms governing pluripotency are conserved from urodele amphibians to mammals [9]. In the lower vertebrate teleosts (medaka, zebrafish, Japanese flounder, carp), Nanog is considered as a maternally inherited pluripotent gene and expressed in gonads in addition to the early development embryos [10,11,12,13,14]. Moreover, Nanog plays a key role in the survival of migrating PGCs during embryonic development [7,15,16]. Furthermore, some germ cells genes, such as *Vasa*, *Dnd*, *Nanos1*, *Igf*, *Pik3*, *Sdf1a-cxcr4b*, and *Puf-A,* have been identified as being involved in PGCs migration in vertebrates [17]. In mice, *Nanog*-knockdown PGCs revealed that a substantial number of genes, such as *Tial1*, *Id1* and *Suz12*, are regulated [16]. In medaka, Nanog regulates PGC migration through *Cxcr4b* [18]. In zebrafish, Nanog suppresses the expression of *Vasa* by directly regulating *nlk1* in the early embryo [19]. However, being distinct from the findings of the studies, a Nanog homolog is missing in anuran amphibians because of chromosomal translocations. Moreover, overexpressing the closest homologs of Nanog (i.e., Vent proteins) in *Xenopus* could not substitute for the function of Nanog, which suggests that the function of Nanog is evolutionarily conserved in maintaining cell pluripotency from bony fishes (teleosts) to mammals [20]. In comparison with their mammalian and teleost, functional identification of Nanog in reptiles remained limited. 

A turtle is an ancient reptile existing on the earth, and is considered as an excellent model for investigations in evolutionary and developmental biology. Chinese soft-shell turtle (*Pelidiscus sinensis*) is the most widely farmed aquatic reptile in China. It is a valuable type of food because of its delicious meat and nutritious yolk. Moreover, the turtle could also be used in traditional medicine. Therefore, it has high nutritional, pharmaceutical, and economic value. Due to the restriction of season, environmental conditions, and overharvesting, wild Chinese soft-shell turtle has been encountered with an extreme decline of populations, it is of great significance to study the germ cells development and proliferation, thus, to improve turtle breeding and maintain its population and resource. Chinese soft-shell turtles are a kind of spring-autumn breeding turtle. Ovulation of the females lasts from May to August, while spermatogenesis of the males begins in May, and reaches maturation in September, extending into November. Specifically, the spermatozoa mature in September, and then leave the testis, entering and being stored in the epididymis throughout the entire year, which is uniquely different from other reptiles. Although oogenesis is initiated at about the same time as spermatogenesis, the time when spermatozoa mature (September) and that of ovulation (May) is mismatched [21]. Generally, the sexual maturation age of Chinese soft-shell turtle is 2–3 years in South China. Our previous study found that the testis of 1-year-old male Chinese soft-shell turtle mainly contains various sertoli cells and a small number of germ cells at early stage. Furthermore, the 2-year-old male testis contains a large number of germ cells, including most stages of germ cells (from spermatogonia to elongated spermatids). When male Chinese soft-shell turtles reach sexual maturation, the testis evacuates spermatozoa which is stored primarily in the male epididymis and recovers partial stem cell characteristics. Thus, the testis of 3-year-old male Chinese soft-shell turtle mainly contains spermatogonia, spermatocytes, and very few spermatids [22]. As for female Chinese soft-shell turtle, the ovary of a 1-year-old mainly contains early-stage germ cells. The 2-year-old ovary contains most stages of oocytes. The ovary of a 3-year-old mainly contains the late-stage oocytes. 

Although germ cell development and vital regulators modulating germ cell development and differentiation, such as *Vasa*, DAZ family genes, have been investigated in Chinese soft-shell turtle [23,24,25], it is still largely uncertain how the germ cells develop and differentiate in turtles. Therefore, to identify and analyze the *Nanog* gene of *P. sinensis* (hereafter referred to as *PsNanog*) would lay a foundation for further studies on the developing pluripotent cells and germ cells in Chinese soft-shell turtle. In this study, we first identified and analyzed the *PsNanog* gene structure and sequence. Secondly, we predicted the potential *cis*-elements in core promoter region and determined the promoter activity. Thirdly, the relative expression levels in different tissues were also examined. Lastly, the cellular localization of *PsNanog* mRNA and protein was also investigated in gonads. The results imply that turtle Nanog is probably also a major regulator of germ cell development and is mainly expressed in gonads, which is similar to that of teleosts and mammals.

## 2. Materials and Methods

### 2.1. Animals and Tissue Collection

Animals and tissue collection was conducted following the previous method [24]. Specifically, fifty-four healthy adult Chinese soft-shell turtles, including nine males and nine females aged 1+, 2+, and 3+ years of age, respectively, were collected from Wealth Xing Industrial Co., Ltd. aquarium farm in Huizhou, South China. All turtles were anesthetized with an intraperitoneal injection of sodium pentobarbital (20 mg/kg, P3761; Sigma-Aldrich, (St. Louis, MO, USA) and killed, and a panel of tissues including heart, brain, liver, spleen, kidney, testis, and ovary were collected and stored in the RNA safer at −80 °C for RNA extraction, and fixed in 4% paraformaldehyde (PFA) for sections. We declared that all animals used in this study were treated humanely and ethically, and we followed all applicable Chinese institutional animal care guidelines.

### 2.2. Cloning PsNanog cDNA Sequence and Its Promoter 

Total RNA was extracted from 1-year-old tissues (heart, brain, liver, spleen, kidney, testis, and ovary) and 2-, 3-year-old gonads (testis and ovary) of Chinese soft-shell turtle using SV Total RNA Isolation System (Promega, Madison, WI, USA). Specifically, the 1-year-old tissues from three male and three female turtles were mixed for one sample as one biological replicate, in total, three biological replicates, and the 2-, 3-year-old testis and ovary from nine males and nine females, respectively, were also mixed for three biological replicates, for RT-PCR and RT-qPCR analysis. Genomic DNA was isolated from the liver of Chinese soft-shell turtle by the phenol-chloroform extraction method. The quantity and quality of extracted total RNA and genomic DNA were examined by 1% agarose gel and Nanodrop 2000. Additionally, 1 μg total RNA was reverse-transcribed *via* SuperScript™ III First-Strand Synthesis System (Invitrogen, Carlsbad, CA, USA) after Dnase treatment. Primers for isolating the Chinese soft-shell turtle *Nanog* cDNA (PsNanog-F/PsNanog-R) and for cloning the *PsNanog* promoter (pPsNanog-F/pPsNanog-R) were designed according to its genome data by Primer 5.0 software, which were shown in Table S1. PCR was conducted under the condition: 94 °C for 1 min, 33 cycles at 94 °C for 30 s, 57 °C for 30 s, 72 °C for 2 min, and 72 °C for 10 min. The PCR products were separated on 1% agarose gel, cloned into a pMD19-T vector and 10 clones were sequenced by Sangon Biotech (Shanghai, China).

### 2.3. Bioinformatic Analysis of PsNanog Gene and Its Promoter 

All nucleotide sequences cloned and their homology across species were analyzed using the BLAST of National Center for Biotechnology Information (NCBI). Multiple sequence alignments were conducted using Vector NTI 11. Moreover, a phylogenetic tree of Nanog proteins was constructed by the method of neighbor-joining (NJ) in MEGA 6.0 and the bootstrap value was set to 1000. Bioinformatic analysis of promoter sequence and potential transcription factor binding sites (TFBSs) within the 5′-regulatory region of the *PsNanog* was mainly performed using the animalTFDB 3.0 website (http://bioinfo.life.hust.edu.cn/AnimalTFDB#!/ (accessed on 12 August 2021)). The potential transcription start site (TSS, +1) was predicted by the Neural Network Promoter Prediction program (NNPP, http://www.fruitfly.org/seq_tools/promoter.html (accessed on 12 August 2021)).

### 2.4. Luciferase Assay

For the luciferase assay, three promoter fragments: PsNanog1 (−1560 − +1), PsNanog2 (−999 − +1), PsNanog3 (−499 − +1), were amplified using primers listed in Table S1, and ligated into the pGL3-basic vector to construct the plasmids, pGL3-PsNanog1, pGL3-PsNanog2, and pGL3-PsNanog3, respectively. Human cells 293T were cultured in an incubator with 5% CO_2_ at 37 °C. With the pRL-TK plasmid (a reporter vector for expressing Renilla luciferase) as the internal control, the three *Nanog* promoter plasmids and pGL3-basic (a reporter vector for expressing firefly luciferase) were transferred into the 293T cells using Lipofectamine 2000 following the kit manual (Invitrogen, cat.no.11668-027). The enzyme activities of LUC were determined using a Dual-Luciferase^®^ Reporter Assay System following the manual (Promega, Cat. No. E1910). The measurements were performed using a luminometer (TECAN, Infinite M1000 Pro, Männedorf, Swiss), each assay was conducted with three technical replicates, in three biological replicates. Data processing was used by Graphpad prism (Graphpad Software, San Diego, CA, USA), and the difference was considered to be at significant and very significant levels at * *p* < 0.05 and ** *p* < 0.01, respectively.

### 2.5. Expression Analysis of Nanog mRNA by RT-PCR and RT-qPCR

To determine the tissue expression profiles of *PsNanog*, RT-PCR, and RT-qPCR were performed by *PsNanog*-specific primers (PsNanog-rt-F/PsNanog-rt-R, Table S1) and *β-actin* (an internal control) primers (β-actin-rt-F/β-actin-rt-R, Table S1). RT-PCR was run for 120 s at 95 °C and followed by 32 cycles of 15 s at 95 °C, 30 s at 60 °C, and 30 s at 72 °C. The PCR products were separated on 2% agarose gels and documented with a bioimaging system (Alpha Innotech). RT-qPCR was run with the following program: 95 °C for 2 min, followed by 40 cycles of 95 °C for 15 s, 60 °C for 30 s, and 72 °C for 30 s, ended with the melt curve 65 to 95 °C, with 0.5 °C/s each temperature lasting for 0.5 s. Analysis was performed using three biological samples, with three replicates. The relative expression levels of *PsNanog* were calculated using 2^−ΔCt^ method. Statistical analysis was calculated by using *Least-Significant Difference* (*LSD*) method in SPSS 20 software (SPSS Inc., Chicago, IL, USA) for multiple comparison, and the difference was considered significant when *p* < 0.05. Data were presented as mean ± standard deviation (SD).

### 2.6. Cryostat Sections of Testis and Ovary

Samples of gonadal tissues were dissected and fixed in 4% PFA at 4 °C overnight, after washing with phosphate buffer saline (PBS), the samples were immersed in 30% saccharose–PBS buffer overnight at 4 °C. Then samples were embedded in O.T.C. (Tissue-Tek), and then testis samples were cut in sections of 4 μm and ovary of 8 μm with a cryostat microtome (Leica). The cryosections were mounted on frost glass slides (4951 PLUS-001E, Fishery, Fort Collins, CO, USA) and stored at −80 °C till use.

### 2.7. In Situ Hybridization

*In situ* hybridization (ISH) on cryostat sections was performed as previously described [26]. The preparation and purification of RNA probes was followed as previously described [24]. In brief, a 1.0 kb cDNA fragment of *PsNanog* was amplified from a Sp6 or T7 promoter by using the digoxigenin RNA Labeling Kit (Roche) for synthesizing sense and antisense probes. The primers sequence for probe synthesis of *PsNanog* was listed in Table S1 (Nanog probe-T7-F/Nanog probe-Sp6-R). Then, the RNA probes were purified with RNase-free TURBO DNase and then LiCl according to the manual of mMESSAGE mMACHINE kit (cat# AM1340, Ambion, Austin, TX, USA). The chemical *in situ* hybridization (CISH) signals were developed with BCIP/NBT substrates on sections, and sections were counterstained by propidium iodide (PI) for nuclear staining.

### 2.8. Preparation of Anti-Nanog Antibody

The monoclonal anti-PsNanog (aPsNanog) was made by a company (China) with the expressed and purified peptide antigen (MSAHLAMPAYQAYPAGVGTGIKYGDYYWNCPGEMDSAPHKEAADADVAVPEPEEKPLPNPELSPASSSSGTLLRYTPDSATSPNAAPPSPHPAIRMGGGGSGGGVKKAKT). In total, 6 positive hybridoma cell strains were screened and obtained by EliSA analysis, and the antibodies were collected from cell supernatant respectively. Then, the specificity of the antibodies, aPsNanog was tested by Western blot with the protein extracted from Chinese soft-shell turtle ovaries. Briefly, 20 µg protein solution of gonads was loaded into lanes, respectively, separated through 10% SDS-polyacrylamide gels, and electroblotted onto polyvinylidene difluoride membranes by an elecotroblotter (Biorad, Hercules, CA, USA). Membranes were blocked with 5% bovine serum albumin (BSA, V900933; Sigma) in TBST buffer (25 mM Tris-HCl, 137 mM NaCl, 2.7 mM KCl, and 0.1% Tween 20, pH 7.4) for at least 1 h at room temperature. After washing with TBST, membranes were incubated with 1st antibodies (1:500 dilution in TBST with 2% BSA) for 2 h, including monoclonal aNanog (homemade, this paper). Then, the membranes were washed with TBST and incubated HRP-conjugated anti-mouse IgG (Boser, Cat. No.BA1050) (1:5000 dilution in TBST) for 1 h at room temperature. After membrane washing, protein blot was developed using the Enhanced HRP-DAB Substrate Colorimetric Kit (Tiangen, China) and imaged with an image system (Tanon-1600, Tanon, China).

### 2.9. Immunofluorescence Staining of Ovary and Testis Sections

PsNanog protein was detected by the monoclonal antibody, aPsNanog in ovary and testis sections through immunofluorescence staining following the previous described [23]. Briefly, gonad sections were rehydrated and blocked with 5% BSA in PBS for 1 h at room temperature, then incubated overnight at 4 °C with the primary antibodies, including aPsNanog, aPCNA [27] (positive control) and normal serum (negative control), the primary antibodies and control serum were diluted 1:100 with 2% BSA in PBS, respectively. On the second day, the sections were washed three times with 0.1% Tween-20 in PBS (PBST), blocked with 5% goat serum in PBS for 1 h, and incubated with the secondary antibody HRP-conjugated anti-mouse IgG (Boster, Cat. No.BA1050) for 1 hr at room temperature. After 3× stringent washes with PBST and PBS, TSA-Plus TMR/Fluorescein System was used to develop signals according to the product manual (NEL756, NEN Life Science). All images were acquired with a confocal microscope (Zesis LSM880). 

## 3. Results

### 3.1. Molecular Characterization of PsNanog and Its Promoter

**Figure 1:**
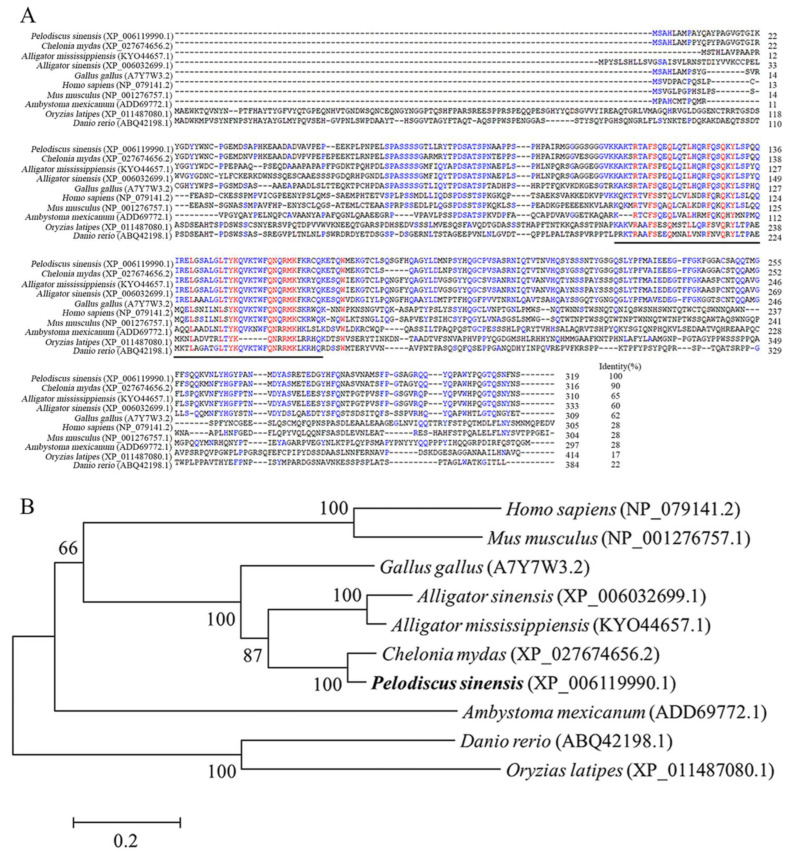
Multiple alignment and phylogenetic analysis of Nanog proteins. (**A**) Multiple alignment of Nanog in different phyla. The conserved homeodomain of 60 amino acids is underlined with a thick line. The identical amino acids are shown in red letters. The species names and GenBank accession numbers are shown to the left, and Identity scores relative to Chinese soft-shell turtle are shown on the end of the alignment. The alignment was generated with Vector NTI 11. (**B**) Phylogenetic analysis of Nanog in different species. The phylogeny tree was constructed by Mega 6.0 using the Neighbor-joining method. The bootstrap value was set to 1000.

According to the Chinese soft-shell turtle genome database and the predicted mRNA sequence (accession number: XM_006119928.3), we designed gene specific primer pair and cloned *PsNanog* from turtle ovary cDNA, containing a 1032 bp full-length cDNA with 21 bp 5′ UTR, 51 bp of 3′ UTR and 960 bp of the open reading frame (ORF, Figure S1), with an identity of 99.9% with the computer-predicted sequence retrieved from NCBI GenBank (accession number: XM_006119928.3; Figure S2). The deduced amino acid (aa) sequence of *PsNanog* contained 319 residues. Similar to Nanog orthologs previously identified in other species, the PsNanog protein contained a highly conserved HD within positions 107-166 (Figure 1A). Multiple alignment of the Nanog protein aa sequences revealed that PsNanog shared a high identity of 90% with that of green turtle (*Chelonia mydas*, XP_027674656.2), a moderate identity of 65%, 60%, 62% with American alligator (*Alligator mississippiensis*, KYO44657.1), Chinese alligator (*Alligator sinensis*, XP_006032699.1), and chick (*Gallus gallus*, A7Y7W3.2), respectively. However, PsNanog shared lower identities at around 28%, with those of human (*Homo sapiens*, NP_079141.2), mouse (*Mus musculus*, NP_001276757.1), and axolotl (*Ambystoma mexicanum*, ADD69772.1), at 22% with zebrafish (*Danio rerio*, ABQ42198.1), at 17% with medaka (*Oryzias latipes*, XP_011487080.1) (Figure 1A). Generally, there are significant divergence in Nanog protein sequence across phyla. A phylogenetic tree was constructed to evaluate the evolutionary relationships between the predicted PsNanog and its orthologs from different species (Figure 1B). The Nanog proteins were divided into two main branches with above 60% bootstrap support, which were mammals, birds, reptiles, amphibians, and tetrapods clade, and bony fishes. In the former, *P. sinensis* showed a tight cluster with *C. mydas*, and then was gradually clustered with *Alligator* and *Gallus*, forming a branch of reptile. In bony fishes, *D. rerio* and *O. latipes* showed a tight cluster. The Nanog of *G. gallus* and *A. mexicanum* were clustered in a branch between mammals and teleosts.

The genomic DNA sequence of *PsNanog* (NW_005854348.1) was obtained from NCBI, a predicted *PsNanog* gDNA fragment of 4200 bp was used by the designing gene specific primers to isolate the *PsNanog* cDNA. A comparison between *PsNanog* cDNA and gDNA sequence revealed that the gDNA fragment contained four exons (i.e., exon 1, 202 bp; exon 2, 269 bp; exon 3, 90 bp, exon 4, 471 bp) and three introns (i.e., intron 1, 1256 bp; intron 2, 734 bp; intron 3, 1178 bp) (Figure S3). All exon–intron boundaries are conformed to the 5′ GT and 3′ AG splicing rule. The *PsNanog* gene exhibited a similar genomic organization to teleost fish and mammalian *Nanog* gene despite their different gene size (Figure S3). In addition, the conserved HD was located between the second and third exons in those *Nanog* genes.

**Figure 2:**
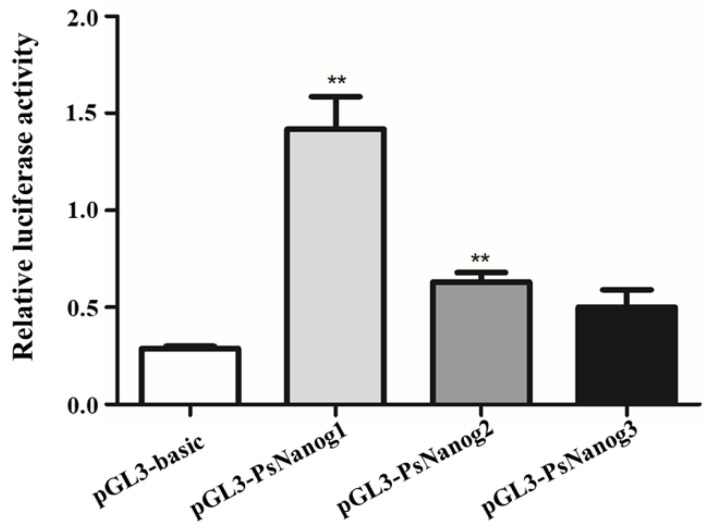
Analysis of *PsNanog* promoter activities in 293T cells. Firefly luciferase expression levels were normalized to the luciferase activity of internal Renilla control and expressed as relative luciferase units. The results were presented as means ± SD. PsNanog1 (−1560 − +1), PsNanog2 (−999 − +1), and PsNanog3 (−499 − +1) were three promoter fragments. ** represents the significant difference (*p* < 0.01).

In order to obtain *PsNanog* promoter regulatory region, we cloned a length of 1870 bp *PsNanog* promoter, containing the 5′ flanking, exon 1, and partial DNA fragment of intron 1 (Figure S4). *In silico* analysis of the promoter region from −1560 to +1 bp, it was found that the regulatory region contained essential binding sites for multiple TFs, including Oct4 (i.e., Pou5f1), Sox2, Nanog, PRDM1, p53, Tcf3, CREB1, Zfp42, STAT3, Wt1, and Sox9. Additionally, binding sites for GATA-1, AP1, and Sp1 were also identified (Figure S4). The potential TSS was located at +1 bp, and the TATA-box was located at −29 bp (Figure S4). To further verify the active region of the promoter, three promoter deletion vectors: pGL3-PsNanog1 (−1560 − +1, 1561 bp in length), pGL3-PsNanog2 (−999 − +1, 1000 bp in length), and pGL3-PsNanog3 (−499 − +1, 500 bp in length) were constructed. The dual luciferase assay showed that the activity of pGL3-PsNanog1 was highest among the three fragments, and it was followed by pGL3-PsNanog2 and pGL3-PsNanog3. The promoter activities of pGL3-PsNanog1 and pGL3-PsNanog2 were both significantly higher than that of pGL3-basic (Figure 2). 

### 3.2. Transcription Expression of PsNanog in Different Tissues

**Figure 3:**
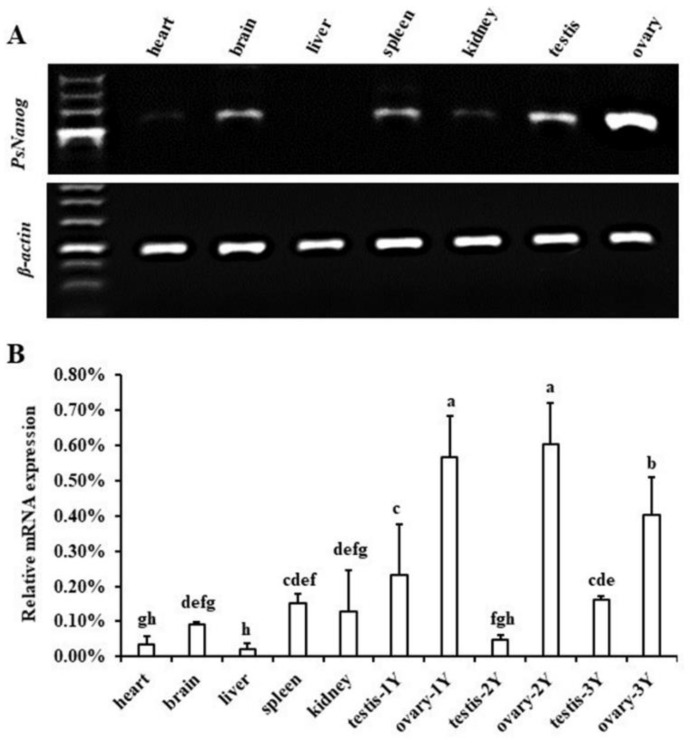
Transcription profile of *PsNanog* mRNA in adult tissues of *Pelodiscus sinensis* by RT-PCR (**A**) and RT-qPCR (**B**). (**A**) The *β-actin* was amplified as the internal control. The adult tissues were used as indicated. The *PsNanog* mRNA is rich in ovary. (**B**) X-axis represents different tissues, including heart, brain, liver, spleen, kidney, ovary and testis of different ages. Y-axis indicates the relative expression levels (%) of genes. *β-actin* was used as normalization factor of RT-qPCR. Data were presented as means ± standard deviations (SD). Different lowercase letters indicated the significant difference at *p* < 0.05 by using the method of *Least Significant Difference* (*LSD*).

The transcription expression level of *PsNanog* was detected by RT-PCR and RT-qPCR in different tissues of Chinese soft-shell turtle. The RT-PCR result showed that *PsNanog* was highly expressed in ovaries, moderately in testis, spleen, brain, and weakly in kidney and heart tissues, but undetectable in liver (Figure 3A). This result was confirmed by RT-qPCR (Figure 3B). In addition, the expression levels of *PsNanog* in ovaries of the 1-, 2-, and 3-year-old animals were significantly higher than that in other tissues, and the expression level of *PsNanog* was higher in ovary than testis of turtle at all ages examined. Specifically, the expression levels of *PsNanog* in ovaries of 1- and 2-year-old tissues were not shown significant differences but they both were significantly higher than that in 3-year-old ovary. However, the *PsNanog* expression pattern in testis of 1- to 3-year-old animals showed a trend of decreasing first and then gradually increasing (Figure 3B).

### 3.3. The Expression and Cellular Localization of PsNanog mRNA in Chinese Soft-Shell Turtle Ovary during Oogenesis and Testis during Spermatogenesis

**Figure 4:**
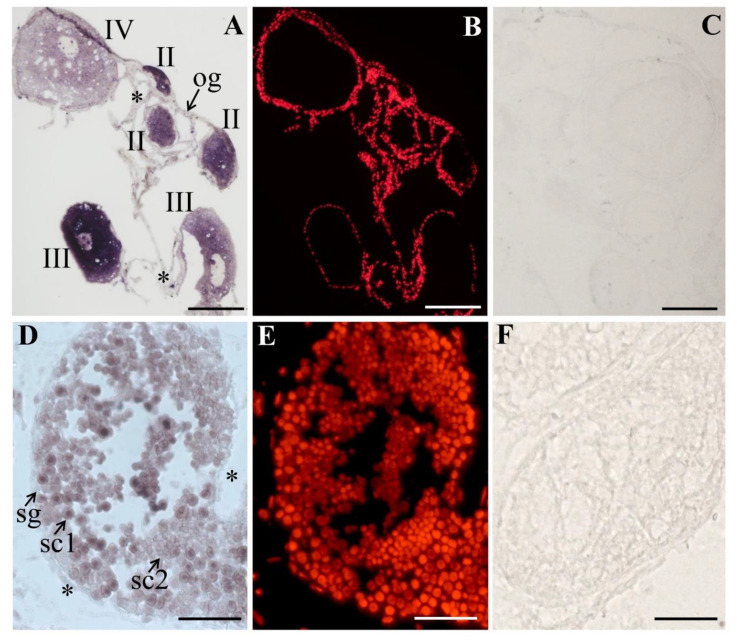
The *PsNanog* mRNA expression in ovary and testis by chemical *in situ* hybridization (CISH). CISH was performed on gonadal sections. The signal was in purple stained by Alkaline phosphatase (AP) (**A**,**D**), and the nucleus was stained with propidium iodide (PI) in red color (**B**,**E**). Sense probe as a negative control had no signals (**C**,**F**). *PsNanog* mRNA was expressed in primary oocytes. In the primary oocytes, the signal was strongest and uniformly distributed in the cytoplasm, and then the signal was concentrated in the cytoplasm of perinuclear region in the growing oocytes and gradually weakened with the growth of oocytes. Og, oogonia; stage II–IV, primary oocytes. *PsNanog* mRNA was expressed in spermatogonia (sg), primary spermatocyte (sc1), secondary spermatocyte (sc2). Somatic cells were labeled with stars. Scale bars, 50 μm.

The process of oogenesis is divided into 10 consecutive stages (i.e., Stage I–X) on the basis of the structures of follicular cells, zona pellucida, and some other related features. The female germ cells are oogonia, primary oocytes (Stage I–V), growth oocytes (Stage VI–IX), and mature oocytes (Stage X) [28]. CISH was performed on ovary tissue sections at various developmental stages, the results showed that *PsNanog* mRNA was expressed only in germ cells. *PsNanog* mRNA signals were strongest in the early primary oocytes (Stage II–IV), and uniformly distributed in their cytoplasm. As the oocyte grew, the signal gradually became weak (Figure 4A,B), while there was no signal detected in the negative control (Figure 4C).

The turtle testis is composed of tubules, lined with seminiferous epithelia containing somatic cells and developing germ cells, such as spermatogonia, primary spermatocytes, secondary spermatocytes, round spermatids, elongated spermatids, and spermatozoa [29]. To examine the cellular distribution of turtle *Nanog* in male germ cells during spermatogenesis, CISH was conducted on the cryostat sections of Chinese soft-shell turtle testis. The nucleus was stained by PI. In the 3-year-old turtle testis (during recovery), *PsNanog* mRNA was exclusively expressed in germ cells. Specifically, *PsNanog* mRNA was expressed in most stages of male germ cells during spermatogenesis: highest in primary spermatocytes, moderate in secondary spermatocytes, weak in spermatogonia, but undetectable in somatic cells (marked with stars) (Figure 4D,E), while there was no signal detected in the negative control (Figure 4F).

### 3.4. The Expression and Cellular Localization of PsNanog Protein in Chinese Soft-Shell Turtle Ovary during Oogenesis and Testis during Spermatogenesis 

**Figure 5:**
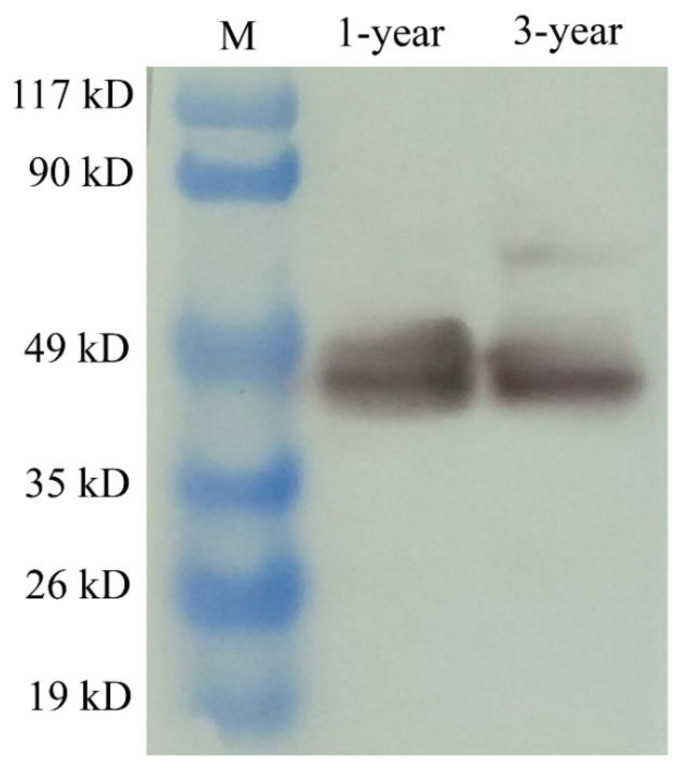
The Nanog protein examined in the turtle ovaries by Western blot. M: protein size markers; 1-year: protein sample from 1-year-old turtle ovary; 3-year: protein sample from 3-year-old turtle ovary. aNanog detected a specific band of around 45 kD in turtle ovaries at different ages.

**Figure 6:**
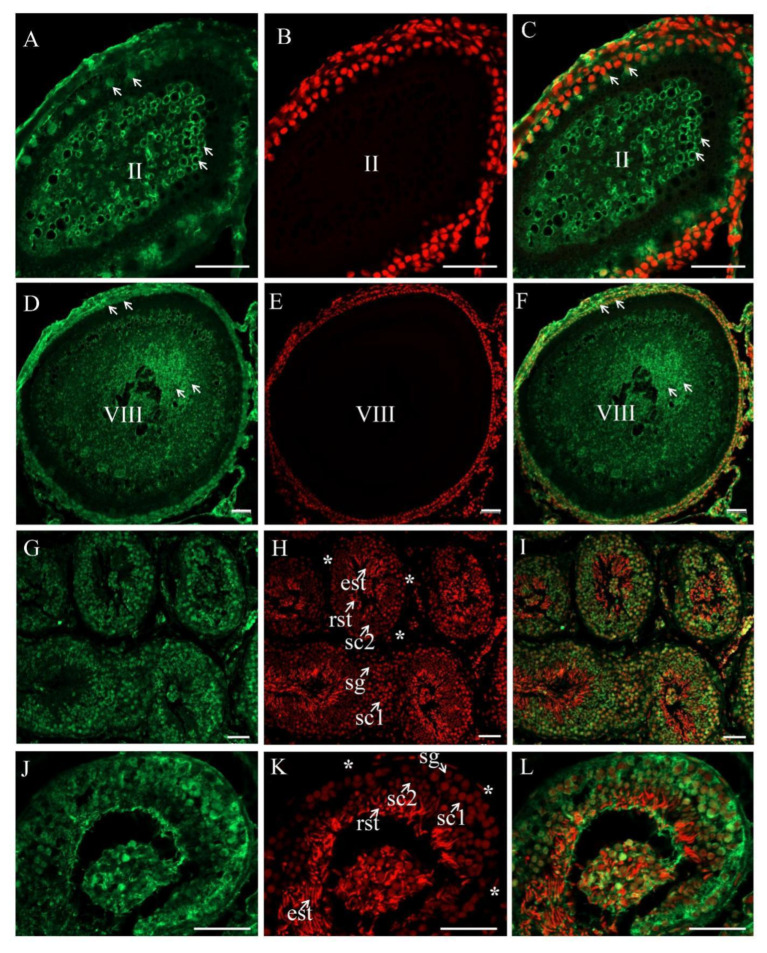
PsNanog protein expression in germ cells. The cellular distributions analysis of PsNanog protein during oogenesis and spermatogenesis. (**A**) Immunostaining was performed on the ovary and testis sections with the antibody aPsNanog (green in **A**,**D**,**G**,**J**), nuclei were counterstained with propidium iodide (PI; red in **B**,**E**,**H**,**K**). (**C**) Merged images of A and B, (**F**) merged images of D and E, (**I**) merged images of G and H, (**L**) merged images of J and K. (**A**–**C**) Stage II oocytes, (**D**–**F**) stage VIII oocytes. PsNanog signals were detected in the cytoplasm and granulosa of Stage II oocytes, while PsNanog was localized in the cytoplasm of the cortical region and granulosa of Stage VIII oocytes (the arrow indicated). PsNanog signals were detected in spermatogonia (sg), primary spermatocytes (sc1) and secondary spermatocytes (sc2), round spermatids (rst), and elongated spermatids (est). Somatic cells were labeled with stars. Scale bars, 50 μm.

In order to detect the cellular distribution of PsNanog protein in Chinese soft-shell turtle germ cells, the homemade monoclonal antibody aPsNanog was used. By Western blot, the specificity of aNanog was examined and a clean band of around 45 kD protein was detected in the turtle ovaries at 1- and 3-year-old, respectively (Figure 5). Meanwhile, the fluorescence immunostaining was conducted on testis sections to examine the specificity and accuracy of aPsNanog (Figure S5). Then, the fluorescence immunostaining was conducted on ovarian sections, the result showed that PsNanog was highly expressed in the cytoplasm and granulosa of Stage II oocytes (Figure 6A–C), while PsNanog was localized in the cytoplasm of the cortical region of Stage VIII oocytes (Figure 6D–F). Intriguingly, it was still highly expressed in granulosa, but the signal became weak in ooplasm in the growing up oocyte (Figure 6D–F). 

Accordingly, the Nanog protein expression was analyzed *via* the immunofluorescence staining on the 2-year-old turtle testis sections, the result showed that the PsNanog protein was majorly expressed in germ cells and highest in the cytoplasm of primary spermatocyte, moderate in the secondary spermatocyte, weak in spermatogonia and spermatids (Figure 6G–L).

## 4. Discussion

### 4.1. Identification of PsNanog Gene

Nanog is first reported in ESCs in 2003. It is a pluripotent stem cell marker, which can maintain the self-renewal and totipotency of ESCs [1,2]). In this study, we cloned a *Nanog* gene from Chinese soft-shell turtle, and its full length was 1032 bp, containing a 960 bp ORF, encoding 319 aa. In mice, the cDNA of *Nanog* is 2184 bp in length and contains an ORF encoding 305 aa [5]. The typical structure of Nanog contains three domains: the variable N-terminal domain, highly conserved HD domain, and the C-terminal domain (W repeats). Specifically, the HD consisting of about 60 aa highly conserved across phyla is the typical characteristic of Nanog protein, and it could bind the downstream DNA and play a vital role in its function. Moreover, Nanog proteins of all species possess the conservative structure of HD. Likewise, PsNanog has also the HD domain in positions 107–166 aa (Figure 1A). However, PsNanog does not have the conserved tryptophan (W) repeats, which exist in the C-terminal region of human NANOG [30]. This is similar to that in chick [7] and axolotl [9]. It has been documented that the W repeats are required for Nanog heterodimer formation in mouse. Moreover, homodimerization of Nanog is critical for promoting pluripotency of ESCs [31,32]. In contrast, the Nanog proteins in amphibians of the order Anura have neither HD domains, nor W repeats [20], indicating that the C-terminal domain is variable as the species evolves. Previous studies showed that Nanog proteins were presented during the evolution from invertebrates to vertebrates [4], although the gene is undetectable in the common ancestor of Anurans [20]. In this study, the results of sequence alignment and phylogenetic analysis showed that aa sequences of Nanog proteins exhibit prominent divergences among species examined until now. Generally, the Nanog proteins were divided into two main clades with over 60% bootstrap support, one is the tetrapods clade, containing mammals, birds, reptiles, and amphibians, the other is the teleosts clade. The identity of Nanog protein was up to 90% between Chinese soft-shell turtle and green turtle, these two species are closest to each other in evolutionary. However, PsNanog showed only 17% homology with medaka Nanog, and they were clustered into different branches. The Nanog of *G. gallus* and *A. mexicanum* clustered in a branch between mammals and teleosts, which was in line with the previous findings [12]. In other words, these results indicated that HD of Nanog had evolutionary divergence in the aa sequence and function domains. Likewise, the low sequence identities among vertebrates also imply that *Nanog* may be a rapidly evolving gene. However, *PsNanog* shows a conserved genomic organization with its orthologs. Specifically, *PsNanog* consists of four exons and three introns, which is similar to that of mammals and teleosts [1,6,7,10,12,20]. These results suggested the probably conserved role of HD and species-specific functions of Nanog protein. 

*In silico* analysis of the promoter region of *PsNanog* showed that the promoter region contained basal core promoter elements such as the TATA-box and CAAT-box, and multiple TFBSs (Figure S4). Similarly, turtles, mammals, and teleosts had a conventional TATA-box right upstream of the putative TSS. However, there is lack of a CAAT-box in mouse *Nanog* promoter [33]. Significantly, Nanog plays a critical role in maintaining the pluripotency as a TF. In this study, we identified several *cis*-elements of significant for pluripotency or ESCs. They are Oct4 (i.e., Pou5f1), Sox2, Nanog, Tcf3, and STAT3, which functioned directly in the ESCs or were involved in the pathway associated with pluripotency [18,33,34,35,36]. Similar to Oct4/Sox2 element existing in the mammalian [33,37], we also found the TFBSs for Oct4/Sox2 located randomly in the upstream regulatory region. However, only the element of Oct4 not Sox2 had been found in all teleosts’ promoter regions [10]. The results indicated that the *Nanog* gene promoters in different species have common regulatory elements, as well as species specific *cis*-elements. Dual luciferase assay showed that the promoter activity of 1561 bp promoter region (−1560 − +1) was stronger than that of 1000 bp promoter region (−999 − +1) and 500 bp fragment (−499 − +1), which may be one Tcf3 and two p53 motif (both known to negatively regulate Nanog expression in mammals: Tcf3 represses its self-renewal function [35], while p53 down-regulates Nanog expression in ESCs during differentiation [36]) were found in the proximal upstream of TSS, and some basic enhancers (AP1 and Sp1) in the distal region, they could regulate the expression of Nanog by interacting with the promoters and enhancers [38]. In *PsNanog* promoter region, three TFBSs for Nanog itself were identified, suggesting that Nanog can not only form a homodimerization in promoting stem cell pluripotency [39], but also be regulated by itself. Additionally, in *Nanog* promoter region, some binding sites of reproduction-related TFs, such as PRDM1, Wt1, and Sox9, were identified, implying the potential role of Nanog in regulating the germ cells development. To address these issues, further experiments are needed.

### 4.2. PsNanog Expression in Different Tissues 

*PsNanog* transcriptional expression in different tissues was detected by RT-PCR and RT-qPCR. Among the tissues examined, *PsNanog* mRNA was majorly expressed in kidney, brain, spleen, and gonads, and the mRNA level in ovary was much higher than that in testis (Figure 3). Thus, *PsNanog* was predominantly expressed in ovary which is similar to the results from fish such as blunt-snout bream, farmed carp, Japanese flounder, goldfish, and medaka [10,12,14,40,41]. However, *PsNanog* was also slightly expressed in the brain and spleen of Chinese soft-shell turtle (Figure 3), which was consistent with fish *Nanog*, including farmed carp, Japanese flounder, goldfish, and medaka [10,12,14,40], different from the expression pattern of mammals *Nanog* gene. The expression of *Nanog* in mammal was not detected in adult tissues in mouse and human [6]. NANOG is undetectable or at very low levels in the majority of postnatal human tissues [42]. Furthermore, mouse Nanog is selectively expressed in stratified epithelia, which could proliferate when overexpressing *Nanog* [43] and the transcripts of human *Nanog* have been detected in adult bone morrow [44]. Thus, the expression of *Nanog* in adult tissues might be related with the stem cell compartment, and Nanog may also be required for germ cells development in fish and turtles. In addition, the *PsNanog* expression in the ovaries of 1- and 2-year-old animals was significantly higher than that in 3-year-old ovary, which might be because 3-year-old female turtles are sexually mature and *PsNanog* expression decreases. However, the expression level of *PsNanog* in testis was weak and then gradually increased as turtle maturation or gonad developing. In *P. sinensis*, the spermatogenesis was almost completed in 2-year-old testis and there were more late stages of germ cells, such as secondary spermatocytes and spermatids in the testis, thus the expression level of *PsNanog* was lowest in turtle testis at 2 years of age. In 3-year-old turtle, the testis is in a recovery status after spermiation, and then the number of early stages of germ cells, including spermatogonia and primary spermatocytes, increases, thus, the expression level of *PsNanog* is increased. Moreover, our study focused on studying the role of *PsNanog* in the development of germ cells in Chinese soft-shell turtle, thus, we used turtles of different ages (including 1, 2, 3 years of age) to analyze the expression levels and distribution of *PsNanog* gene products in different stages of germ cells in gonads. Therefore, to demonstrate the association between the expression profiles of *PsNanog* gene products and the reproductive cycle of Chinese soft-shell turtle, more extensive investigations are needed in future.

### 4.3. PsNanog mRNA Expression in Chinese Soft-Shell Turtle Germ Cells 

Although the *Nanog* gene is reported in most vertebrates, including human, primates, chicken, lizard, and fish, its functions in maintaining pluripotency and especially in the germ line have yet to be elucidated in numerous species [31]. Here, we extensively investigated the expression of *Nanog* gene in the germ cells of adult turtles. The results of CISH showed that *PsNanog* mRNA was mainly distributed in early primary oocytes (stage II–IV), which was similar to that in ovaries of blunt-snout bream [41]. Moreover, *PsNanog* mRNA was not detected in oogonia of Chinese soft-shell turtle, but it was distributed in oogonia of blunt-snout bream and Japanese flounder [12,41]. In zebrafish, *Nanog* mRNA was expressed in the cytoplasm of stage I–II oocytes, and was less expressed in stage III and IV oocytes [13]. In medaka and Japanese flounder, *Nanog* mRNA expression was detected in the previtellogenic oocytes [10,12]. Collectively, the expression of *PsNanog* mRNA was detected in the cytoplasm and granulosa of early oocytes and the cytoplasm of the cortical region of growing oocytes of the ovary. In addition, *PsNanog* mRNA was mainly expressed in the primary spermatocytes, and weakly in secondary spermatocytes (Figure 4D–F), which is similar to that in testis of blunt-snout bream [41], different from that in testis of zebrafish, medaka, and Japanese flounder, in which *Nanog* mRNA was expressed in spermatogonia [10,12,13].

### 4.4. PsNanog Protein Expression in Chinese Soft-Shell Turtle Germ Cells 

Immunohistochemical detection proved that PsNanog was highly expressed in stage II oocytes, as well as being localized in cytoplasm of the cortical region in stage VIII oocytes, which is similar to that of zebrafish Nanog protein in oocytes [13]. In medaka, Nanog protein expression was detected in the previtellogenic oocytes [10,12]. However, the PsNanog protein was expressed in the nuclear of cultured female germ stem-like cell (PSO1) as reported in our previous study [27]. In contrast, it was reported that meiotically differentiating female germ cells do not express detectable levels of Nanog in mouse [15]. Furthermore, chick Nanog is majorly expressed in PGCs and epiblastic cells in the early embryo and thereafter in germ cells [7,8,45]. Additionally, in this study, PsNanog protein was mainly expressed in the primary spermatocytes, and weakly in secondary spermatocytes, spermatids, and spermatogonia (Figure 6G–L), which is similar to the expression of Nanog in blunt-snout bream testis [41]. Additionally, Nanog protein was barely detected in the spermatogonia of chick [8] and mouse [15]. However, the expression pattern of PsNanog is distinct from that in testis of zebrafish and medaka where Nanog protein was expressed in the nuclei of spermatogonia and the cytoplasm of spermatocytes [10,13]. In addition, Ol-Nanog expression patterns in the adult gonads of medaka are similar to those observed for the *Olvas* gene, the homolog of Vasa which is expressed specifically in the PGCs and is necessary for gonad development [46]. Likewise, the expression profile of PsNanog is also similar to those of PsVasa [23]. Thus, *PsNanog* mRNA and protein expression in both male and female gonads suggested that PsNanog plays a key role in differentiation of germ cells, and this function would be conserved to a certain degree between turtle and fish. Together, the expression of the *Nanog* gene in germ cells has been reported in rodents, primates, chicken, some teleost, and axolotl [8,9,15,18,47]. Thus, Nanog may play an important role in germ cell development, but how and when it functions during germ cells development still needs to be clarified in different species. Moreover, heterogeneously expressing *Nanog* has been extensively reported in ESCs and induced pluripotent stem cells (iPSCs) [1,2]. In the bovine embryo, *Nanog* is necessary for inducing the epiblast formation and maintaining cells’ pluripotency [48]. In some endangered wild cats, *Nanog* is essential for inducing their dermal fibroblasts to iPSCs together with overexpressing human pluripotent TFs Oct4, Sox2, Klf4, and cMyc [49]. In this study, *PsNanog* was expressed in different tissues or gonad cells of Chinese soft-shell turtle and the sequence characteristics of *PsNanog* has been identified, with these tissues expressing *PsNanog* and the *PsNanog* gene might be potential factors which would affect Chinese soft-shell turtle germ cell proliferation and differentiation. Moreover, our previous study successfully established an ovarian cell line of Chinese soft-shell turtle, which proved that the ovarian cell line could be isolated for propagation under experimental conditions and easily transfected with plasmid [27]. Thus, with this cell line, the function analysis of *PsNanog* will lay a solid foundation for illustrating the germ cell development and facilitating the genetic resource preservation in turtles.

## 5. Conclusions

In summary, in this study, the full-length cDNA sequence and promoter of *PsNanog* gene was identified and characterized in *P. sinensis*. By sequence alignment and phylogenetic analysis, the PsNanog has a highly conserved HD domain in N-terminal, but it lacks of W repeats in C-terminal compared with other animals, including teleosts, reptiles, and mammals. The RT-PCR and RT-qPCR results showed that the *PsNanog* is expressed in gonads and is richest in ovary. PsNanog is distributed in cytoplasm of early oocytes and granulosa surrounding the oocytes, as well as in the cytoplasm of the cortical region cytoplasm of growing oocytes in the ovary. Likewise, PsNanog is expressed mainly in the cytoplasm of spermatocytes in testis, which is slightly different from that of Nanog in fish and mammals. Collectively, this is the first report on the gene structure and expression of *Nanog* in turtle, which may lay a foundation for further functional analysis of the *Nanog* in turtle, even in the reptiles. Further, PsNanog probably has the conserved function in regulating cell pluripotency and germ cell development in turtle, as in other species, including teleosts and mammals. However, the expression profile of turtle *Nanog* in germ cells at different stages is different from that of Nanog in teleosts and mammals. In a word, the findings of our study proved that PsNanog would play conserved roles in maintaining cell pluripotency and germ cells development, but it may function through different mechanisms in germ cells at a certain stage. This would pave the way for further developing related techniques for manipulation of stem cell and germ cells in turtle.

## Data Availability

All data are available upon request from the corresponding author.

## Supplementary Materials

The following supporting information can be downloaded at: https://www.mdpi.com/article/10.3390/biology11091342/s1, Figure S1: Nucleotide sequences and encoded amino acid sequences of Nanog in *Pelodiscus sinensis*; Figure S2: The sequence alignment between cloned and predicted *PsNanog* gene; Figure S3: Genomic organization of Nanog gene in vertebrate; Figure S4: The promoter sequence of *PsNanog* gene; Figure S5: Fluorescence immunostaining detected the specificity and accuracy of aPsNanog; Table S1: Sequences of primers used in this study.
