# Supplementary material for: The Divergent and Conserved Expression Profile of Turtle Nanog Gene Comparing with Fish and Mammals
Source: Biology (Basel). 2022 Sep 12;11(9):1342. doi: 10.3390/biology11091342 (PMC9495436; doi:10.3390/biology11091342)
Supplement: Supplementary file 1 [file biology-11-01342-s001.zip › Supplementary_Materials.pdf]

SUPPLEMENTARY MATERIALS

The divergent and conserved expression profile of turtle *Nanog* gene comparing with fish and mammals

Kaili Chen[1], Jianfei Xu[1], Wenzhuo Ban[1], Jiaming Tian[1], Zhiming Tan[1], Luo Lei[2], Wei Li[2], Xinping Zhu[2] and Hongyan Xu[1]*

[1] Key Laboratory of Freshwater Fish Reproduction and Development, Ministry of Education, Key Laboratory of Aquatic Sciences of Chongqing, College of Fisheries, Southwest University, Chongqing 402460, China

[2] Key Laboratory of Tropical & Subtropical Fishery Resource Application & Cultivation of Ministry of Agriculture and Rural Affairs, Pearl River Fisheries Research Institute, Chinese Academy of Fishery Sciences, Guangzhou, 510380, China

* Correspondence: xuhyzqh@163.com

1 Supplementary Figures S1 to S5.

2 Supplementary Tables S1.

```
                                                                                                               GTTCCCCAAACACAAACACTC 21

22  ATGAGTGCCCACCTGGCAATGCCCGCCTACCAGGCTTACCCCGCCGGGGTAGGAACTGGAATCAAGTATGGAGACTACTACTGGAACTGCCCGGGCGAGATGGACAGTGCCCCCCACAAA 141
1    M  S  A  H  L  A  M  P  A  Y  Q  A  Y  P  A  G  V  G  T  G  I  K  Y  G  D  Y  Y  W  N  C  P  G  E  M  D  S  A  P  H  K  40

142 GAGGCAGCAGACGCTGATGTGGCGGTGCCGGAGCCCGAGGAGAAGCCCCTCCCTAACCCAGAGCTCTCGCCCGCTTCCTCCAGCTCGGGCACGCTCCTGCGCTACACCCCGGATTCGGCC 261
41   E  A  A  D  A  D  V  A  V  P  E  P  E  E  K  P  L  P  N  P  E  L  S  P  A  S  S  S  S  G  T  L  L  R  Y  T  P  D  S  A  80

262 ACCAGCCCCAACGCCGCGCCCCCATCCCCACACCCTGCGATCCGCATGGGCGGCGGGGGAGCGGAGGAGGGGTGAAGAAAGCCAAGACCCGCACGGCCTTCTCTCAGGAGCAGCTGCAA 381
81   T  S  P  N  A  A  P  P  S  P  H  P  A  I  R  M  G  G  G  G  S  G  G  G  V  K  K  A  K  T  R  T  A  F  S  Q  E  Q  L  Q  120

382 ACCCTGCACCAGCGGTTCCAGAGCCAGAAATACCTCAGCCCCCAGCAGATCCGCGAGCTGGGCTCAGCCCTGGGGTTGACTTACAAGCAGGTAAAGACATGGTTTCAAAACCAACGGATG 501
121  T  L  H  Q  R  F  Q  S  Q  K  Y  L  S  P  Q  Q  I  R  E  L  G  S  A  L  G  L  T  Y  K  Q  V  K  T  W  F  Q  N  Q  R  M  160

502 AAGTTTAAACGATGCCAGAAGGAAACACAGTGGATGGAAAAAGGGACATGCCTATCCCAAAGTGGCTTTCATCAGGCAGGTTACCTGGACATGAACCCCAGTTACCACCAGGGTTGTCCT 621
161  K  F  K  R  C  Q  K  E  T  Q  W  M  E  K  G  T  C  L  S  Q  S  G  F  H  Q  A  G  Y  L  D  M  N  P  S  Y  H  Q  G  C  P  200

622 GTTAGTGCCAGCAGGAACATCCAGACTGTGACCAATGTGCATCAGAGCTATAGTAGCAGTAACACCTATGGGAGTGGCCAGAGCCTGTGTCCCTTCATGGCTATTGAGGAGGAGGGGTTC 741
201  V  S  A  S  R  N  I  Q  T  V  T  N  V  H  Q  S  Y  S  S  S  N  T  Y  G  S  G  Q  S  L  C  P  F  M  A  I  E  E  E  G  F  240

742 TTTGGGAAACCCGGGGGGGCCTGCAGTGCCCAACAGACAATGGGCTTCTTCAGCCAGCAAAAGGTGAACTTATATCATGGCTACCCAGCAAACATGGATTATGCCAGCAGGGAGACAGAA 861
241  F  G  K  P  G  G  A  C  S  A  Q  Q  T  M  G  F  F  S  Q  Q  K  V  N  L  Y  H  G  Y  P  A  N  M  D  Y  A  S  R  E  T  E  280

862 GATGGCTACCACTTCCAGAATGCCTCTGTCAATGCGATGTCCTTCCCAGGCTCTGCGGGGCGCCAGCAGTACCAGCCAGCATGGTACCCTCAAGGGACACAAAGCAACTTTAACTCTTAG 981
281  D  G  Y  H  F  Q  N  A  S  V  N  A  M  S  F  P  G  S  A  G  R  Q  Q  Y  Q  P  A  W  Y  P  Q  G  T  Q  S  N  F  N  S  *  319

982 ACCTGTACTTTCCTTTTTCCTTCCCTCTCCCACTGTTTCTGCCATTCCTCT
```

Figure S1. Nucleotide sequences and encoded amino acid sequences of *Nanog* in *Pelodiscus sinensis*. The 60 residues conserved homeodomain is underlined with heavy line.

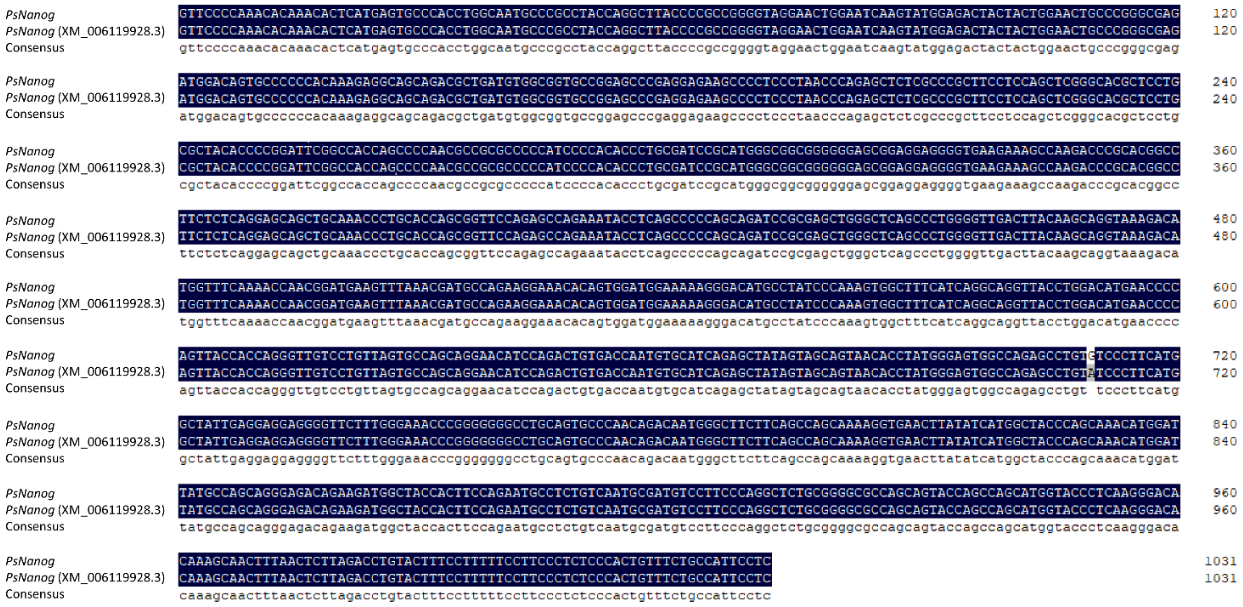

Figure S2. The sequence alignment between cloned and predicted *PsNanog* gene. The first one is cloned and the second is predicted.

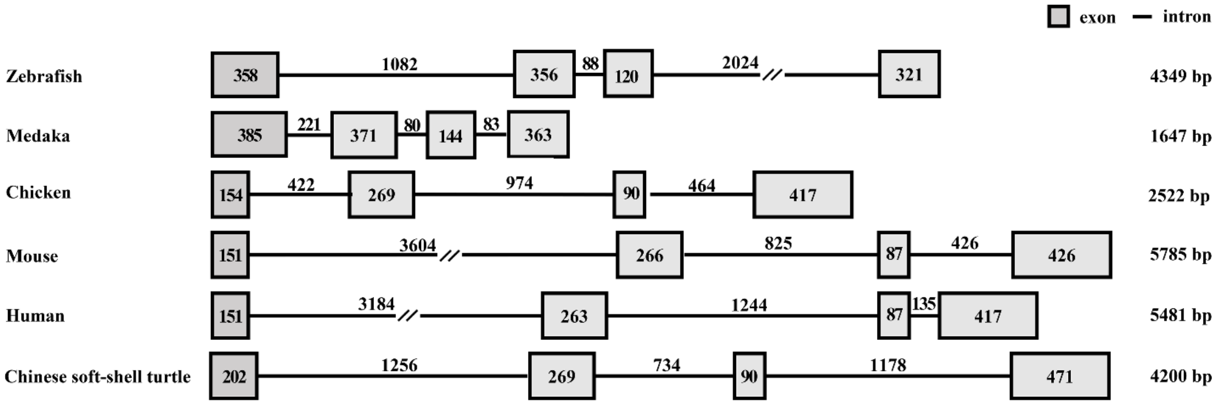

Figure S3. Genomic organization of Nanog gene in vertebrate. The numbers refer to sizes (bp) of the exons and introns. Exons are shown in dark box while introns are shown in straight line. Gene accession numbers: Human, NC_90012.12; Mouse, NC_000072.6; Chicken, NC_Op3088.24, Zebrafish, NC_007135,6; Medaka, NC_019878.1; Chinese soft-shell turtle, NW_005854348.1.

```
                                                                 Wt1      AP1
-1560  aatgatgggaaaactgaggttgaatttaccccactggggtacacagcaagttagtgggagggcactgactaaaacctcaggcttctgacatccaacctat
              Sp1                    GATA-1                     Sox2
-1460  ctatgggtccttgccacaggatcatcctgcttttgaaacatgataatactgtgactgactcactactactgtttataaactgctttgaaaccctcagagg

-1360  atatccccaagaggatatcaacacactttataaacactagtggcagcttccaaatgccacatggcttagttgtgtagcattatatcttcctacctatggg
                                      Oct4/Pou5f1
-1260  aaagacctgaattagcttccatagcaaatcattagcagaaccatgaaaagaaagatctccgggttggaggtctgtggctgaactacaggactctctgacc
-1160  tggggctatttcagtagttctcaaacttcttggcccatagctcacacctctccacgcaagcctttctatggaccatcagctaatcacacttgatgacaaa
                   Zfp42
-1060  atgggtgcagtaggggaggaggcagggtccttgtatcacctcatccaatctaaacaggccagcaacagttcacagccaagaaaatgctcatgtacacaga
                                                                            Wt1
 -960  tgtgcctctgacaaagtgactgctacagccagggatcccctcccagactctactggtctccctcccactgcctccccatatggtgtatctatagttccta
         Sox9
 -860  acagaacaatacaacaaaaatgtctctaatgaccattcattcatatatgagcagagagcctatatctacaggcctgccctgcaaaagtggaccttggtgt
                                                                              STAT3                Nanog
 -760  cgacagggatttcccctaagttcagggctttggcatctggcactagcagactggccaggaccettcctggcacttctcagatggccccatcaagtgccta
                                PRDM1                                                 CREB1
 -660  tcaaaagagaaatttaatctgaaggaagaaaaactttccctctcccctggtttctagcctgagtcactgttgtgctgctcctagtgtcatcacttccccc
                                                              CREB1
 -560  agcaccgagggtaaccagtgctgccattctgacctggttgcattttccctgagctgttgtcactgtgtttacaaactgcaggacagttgggaagcaagcc
           Sp1                                                     Tcf3
 -460  ctctgcttgcaagaccccctttgactggggtttggtttgcctcctatagaattatatgggacaggtgtgtagttcagcttcagaactgaaggcttccaca
                                                                   p53            Oct4/Pou5f1      AP1
 -360  gtctggaggggaggagggaaggttggctggggcagtgaatatttcccacctagggtctcctttctgggatgcttttaacatgtcaggcttgagtcagtg
                             GATA-1                        Oct4/Pou5f1
 -260  ggaagttttacctggacttccccaggctttggatctggcccatgctagggtggactatagaatgccaggaattttggagacattttttaatcctgatgcc
                   p53                         CAAT-box          Nanog
 -160  attgtcgctttcctccccccaccctctccttctctcacaccctccccaccaatcatgctctttaacatgacaatgtgtactgaagacctGGCTGAGGCAGG
                                               TATA-box                                 +1 TSS
  -60  ATATTGTACTCCTCAGAAGGGAACTCCTAGCTATAAAGGTAACTCCCCTAGACAGTCTGAGCACCTTCATCTCTGGGTGCTGAGCTGTTTCCCCCGAAGG
  +41  GAAGTGCAGGCTATTGGGAGGTGGGGGGAGCACCCCTTTAAGCAAGCCCCCAATGAGTGCTCACTTGGCGATGCCCCTTTACCAGCCTTACCCAAGTGGG
 +141  ACTGGCATGAGCTATGGGGAGTTTTACTGGAACTCAGCGGGGGGCACCGAACGTGCAGCATCATGGCCAGGAAGGGGAGCAGTAGCAGCTGGTGATGCCC
 +241  AGTTTCCGGAGGAGGAAAGGCAGAATCCAGgtaagccatcaccgttaagtgagaggagtgaggttcagga
```

Figure S4. The promoter sequence of *PsNanog* gene. The potential transcription start site (TSS, +1) was predicted by the Neural Network Promoter Prediction program (NNPP) is highlighted in bold font. The sense-strand from −1560 to +1 bp of the *PsNanog* promoter was analyzed by animalTFDB 3.0 onine website (http://bioinfo.life.hust.edu.cn/AnimalTFDB#!/). The putative core promoter elements: TATA-box and CAAT-box are boxed with a black line.The regulatory region contained numerous binding sites for multiple transcription factors, including Oct4/Pou5f1, Sox2, Nanog, PRDM1, p53, Tcf3, CREB1, Zfp42, STAT3, Wt1, and Sox9. Additionally, some basal binding sites for GATA-1, AP1 and Sp1 were also identified. AP1: Activator protein 1. CREB1: cAMP Responsive Element Binding Protein 1. GATA-1: GATA-binding factor 1. Nanog: Homeobox Transcription Factor Nanog. p53, p53 tumour suppressor. Pou5f1, POU domain, class 5, transcription factor 1. PRDM 1: PR domain zinc finger protein 1. Sp1: Specificity protein 1. Sox-9: SRY (sex-determining region Y) box 9. STAT3: Signal transducer and activator of transcription 3. Tcf3, T-cell factor-3. Wt1, Wilms' tumor 1. Zfp42: Zfp42 Zinc finger protein.

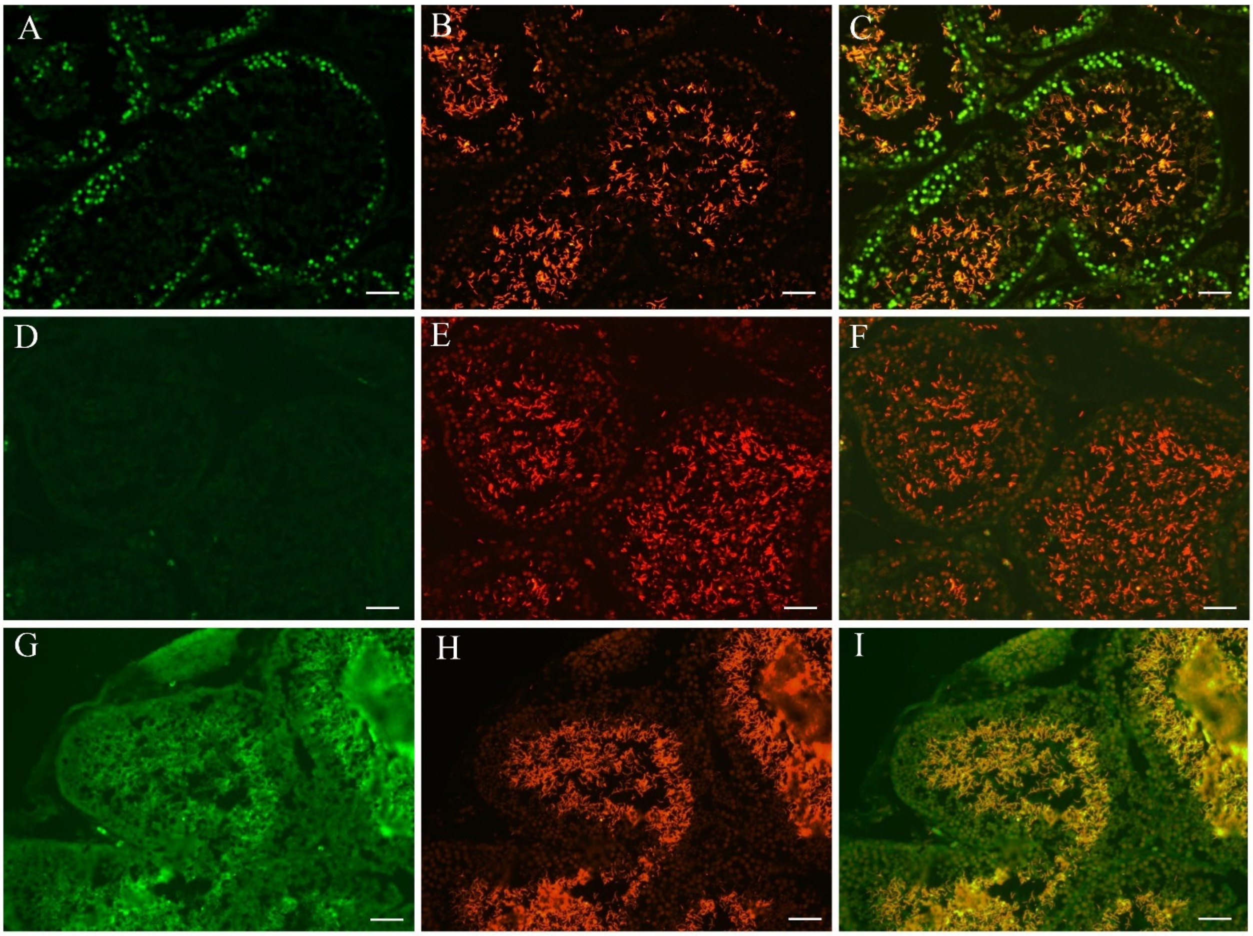

Figure S5. Fluorescence immunostaining detected the specificity and accuracy of aPsNanog. Immunostaining was performed on the testis sections with antibody against PCNA for the positive control (green in A), nuclei were counterstained with propidium iodide (PI; red in B). (C) merged images of A and B. Immunostaining was performed on the testis sections with normal serum for the negative control (there is no signal in D), nuclei were counterstained with PI (red in E). (F) merged images of D and E. Immunostaining was performed on the testis sections with antibody against PsNanog (green in G), nuclei were counterstained with PI (red in H). (I) merged images of G and H. Scale bars, 50 μm.

Table S1. Sequences of primers used in this study

primer	Sequence (5′-3′)	Tm (℃)	Product length (bp)	Experiment
PsNanog-F	GTTCCCCAAACACAAACACT	56.4	1032	Isolation of *Nanog* cDNA
PsNanog-R	AGAGGAATGGCAGAAACAGT	56.4		
PsNanog-rt-F	TTCTTCAGCCAGCAAAAGGT	56.4	244	RT-PCR and RT-qPCR of *Nanog*
PsNanog-rt-R	AGGAATGGCAGAAACAGTGG	57.1		
Psβ-actin-F	AAAGGGAAATTGTGCGTGAC	56.4	197	Internal control of RT-PCR RT-qPCR of *Nanog*
Psβ-actin-R	TTCCATACCCAGGAAGGATG	58.4		
pPsNanog-F	TAGGGAAGATAGGTTTTTTTATAGG	59.2	1870	Isolation of *Nanog* promoter
pPsNanog-R	CAACACCACTAACTAAACTTTTAAC	59.2		
pGL3-psng3-F	TTCTCTATCGATAGGTACCACTGTGTTTACAAACTGCAGGACAGT	64.6	500	constructing pGL3 vectors
pGL3-psng2-F	TTCTCTATCGATAGGTACCGCAACAGTTCACAGCCAAGAAAATG	64.1	1000	
pGL3-psng1-F	TTCTCTATCGATAGGTACCAATGATGGGAAAACTGAGGTTGAATTTAC	64.6	1561	
pGL3-psng-R	AGTACCGGAATGCCAAGCTTCTCAGACTGTCTAGGGGAGTTAC	64.6		
Nanog probe-T7-F	TAATACGACTCACTATAGGGCGGTTCCCCAAACACAAACACT	56.4	1032	*in situ* hybridization
Nanog probe-Sp6-R	TTTAGGTGACACTATAGAATACTAGAGGAATGGCAGAAACAGT	56.4		
