# Supplementary material for: The Divergent and Conserved Expression Profile of Turtle Nanog Gene Comparing with Fish and Mammals
Source: Biology (Basel). 2022 Sep 12;11(9):1342. doi: 10.3390/biology11091342 (PMC9495436; doi:10.3390/biology11091342)
Supplement: Supplementary file 1 [file biology-11-01342-s001.zip › Supplementary_Materials_ original picture.pdf]

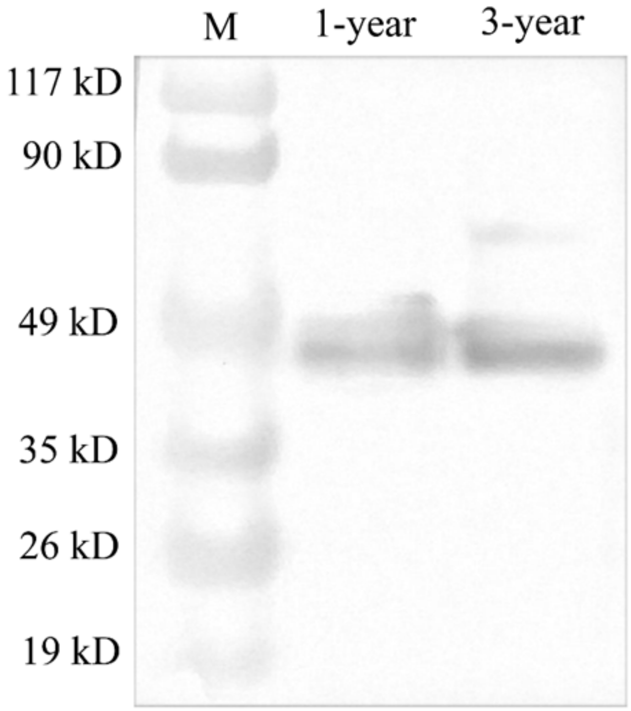

The densitometry readings/intensity ratio of sample 1-year = 112141.36

The densitometry readings/intensity ratio of sample 3-year = 83952.41

The densitometry readings/intensity ratio of each band of Figure 5

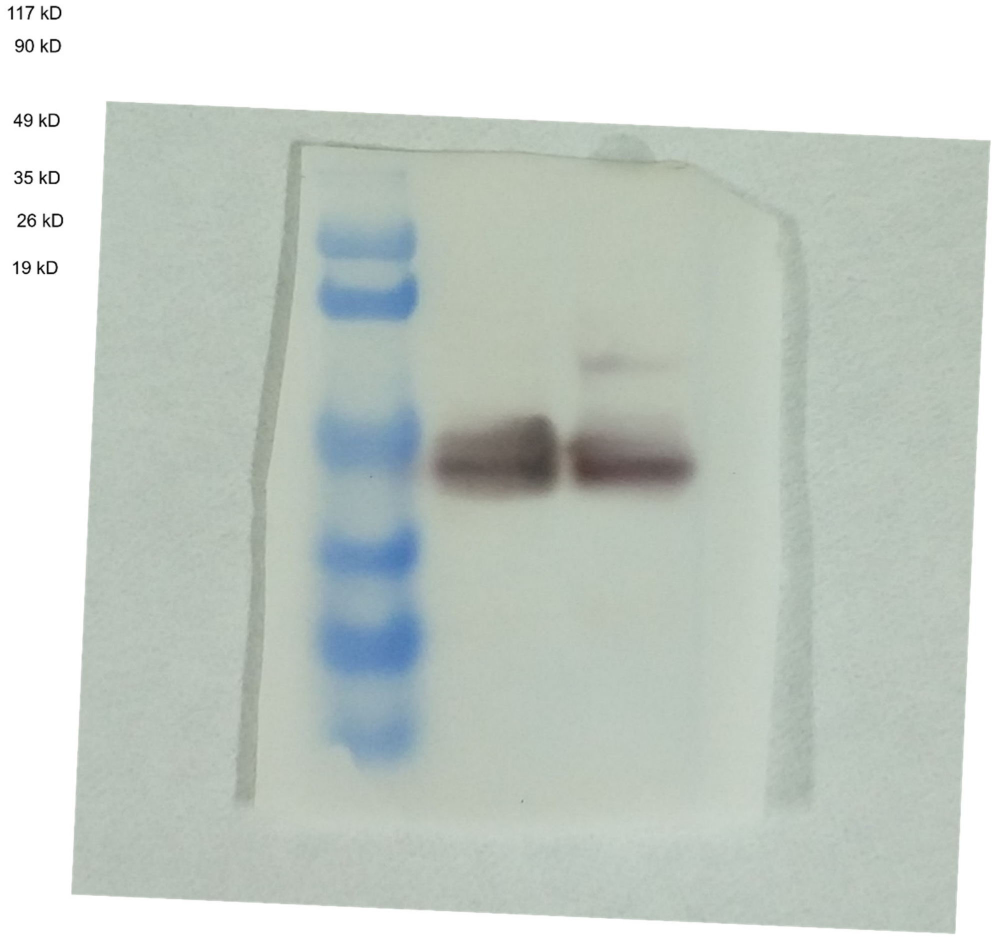

The original picture of Figure 5.

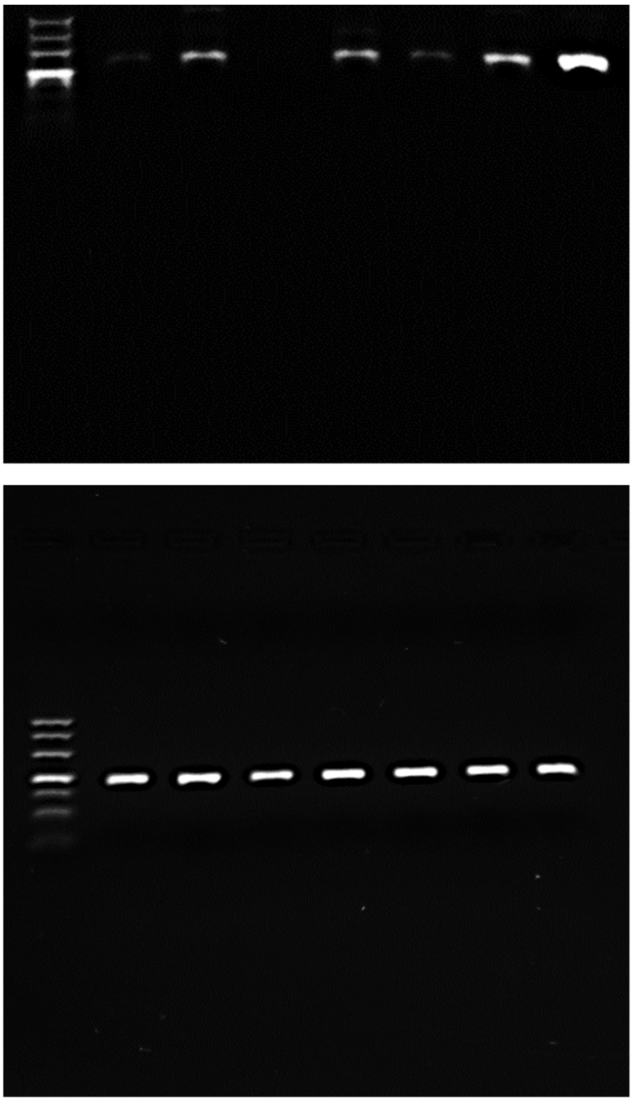

The original picture of Figure 3A.

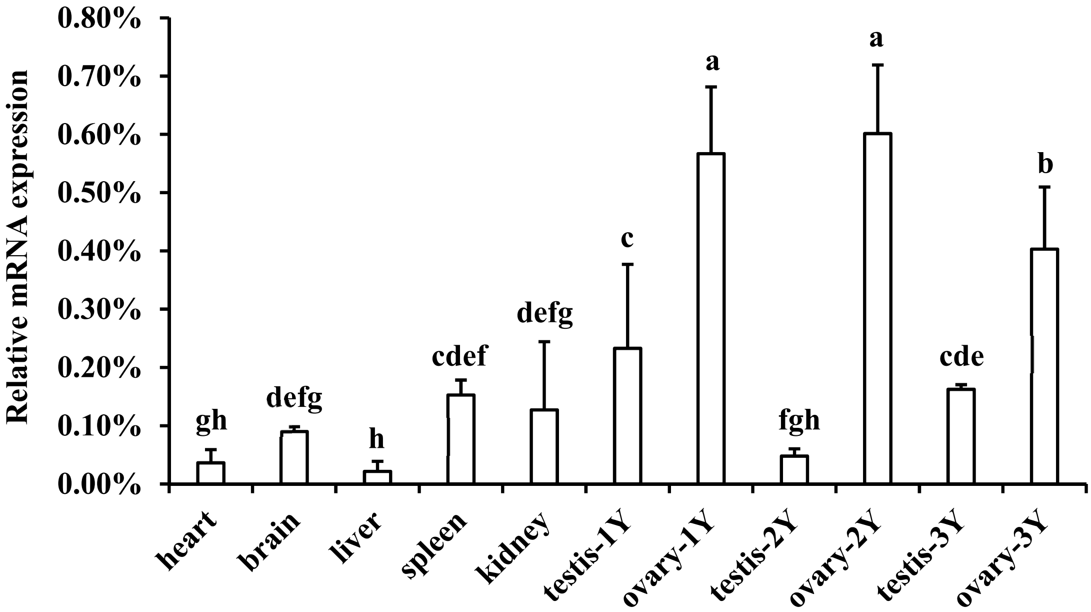

The original picture of Figure 3B.
